# A life-cycle approach to digital health technology assessment

**DOI:** 10.1017/S0266462326104097

**Published:** 2026-08-10

**Authors:** Ashwini Kanakapura Sriranga, Baptiste Vasey, Mateo Campos, Miguel Ángel Armengol de la Hoz, Ruben Casado Arroyo, Stephen Gilbert, Rebecca Mathias, Lucia Osoro, Peter McCulloch

**Affiliations:** 1 Nuffield Department of Surgery, University of Oxford, UK; 2 Division of Thoracic and Endocrine Surgery, Geneva University Hospitals, Switzerland; 3 Data Science Lab, Fundación Pública Andaluza Progreso y Salud MP, Spain; 4 Department of Cardiology, Université Libre de Bruxelles, Belgium; 5 Else Kröner Fresenius Center for Digital Health, Dresden University of Technology, Germany

**Keywords:** health technology assessment, life-cycle evaluation, evidence generation

## Abstract

The approach of Health Technology Assessment, which developed from the Evidence-Based Medicine movement of the 1990s, has proved its value in appraising the value of pharma products but presents methodological challenges when applied to medical devices and digital technologies, whose importance has increased enormously during the last 30 years. The concept of a single assessment, which can summarize relative effectiveness and cost-effectiveness, has been further challenged by the rapidly rising importance of software-driven devices, and latterly Artificial Intelligence. The need to adopt a broader evaluation approach based on product life cycles has been recognized by both regulators and HTA bodies, but practical frameworks for this have not yet been widely adopted. We describe a Life-Cycle–Based Evaluation (LCBE) approach to the assessment of Digital Health Technologies, developed as part of the ASSESS-DHT Horizon Europe 2020 project, EU project 101137347. The LCBE framework is currently being evaluated in pilot studies and aims to support early, proportionate, and continuous assessment and foster collaboration among developers, regulators, and HTA bodies to ensure timely, rigorous, and adaptive evaluations across the technology life cycle.

## Introduction

Health Technology Assessment (HTA) has become a key step in the evaluation of health interventions. It guides decision-making based on comparative clinical efficacy, safety, and cost-effectiveness, as well as ethical, social, and organizational implications (1;2). While HTA has proved highly valuable in evaluating pharmaceuticals, its methodologies have not proved as well suited to the challenges of nonpharmaceutical interventions (3;4). Medical devices, especially low and intermediate risk ones, require far less clinical evidence than drugs to pass the regulatory hurdles in both the EU and US jurisdictions, and the marketing of devices that do not have adequate clinical evidence for evaluation of cost and relative effectiveness is therefore commonplace. The Medical Device Regulation (MDR) published by the European Union in 2017 was intended to reduce this evidence deficit, but has only been partially successful, arguably because it did not provide any specific guidance on evaluation methodology. The advent of Digital Health Technologies (DHTs) presents further new and profound challenges to existing HTA frameworks (5), particularly because they routinely undergo frequent or continuous software modification after achieving market access HTA organizations such as NICE have begun to address this problem through their recently announced *life-cycle approach* to digital and AI technologies, but at present, no overarching methodological guidance on how DHTs should be evaluated at different life cycle stages has emerged (6). The rapidly increasing importance of DHTs incorporating Artificial Intelligence (AI), with its capacity for learning and autonomy, has made this both an evaluation gap and an evidence generation gap. Evidence gaps arise principally because there is insufficient funding or incentive for manufacturers to generate the necessary evidence. HTA frameworks must therefore adapt to assess diverse forms of evidence across the technology life cycle, while payers and manufacturers develop mechanisms to fund robust evidence generation at each life-cycle stage. This opinion piece argues that one-time, static health technology assessment is no longer fit for the purpose for digital health technologies, and that a staged, life-cycle–based approach is now required.

Digital technologies are dynamic, and they can and do evolve rapidly post-market, either through algorithmic self-modification of AI devices, or through frequent software updates (7). This property makes reliance solely on a single HTA assessment insufficient, highlighting the need for structured reassessment and life-cycle evaluation. A modernized approach is therefore required, which spans the entire life cycle of the technology, accounts for its evolving characteristics (8), and aligns with the needs and objectives of regulators, HTA bodies, and payers (9). Collaborative responses such as the UK’s pairing of NICE AI and the Digital Regulations Service are beginning to appear, but they do not, as yet, encompass the needs of the whole life cycle in a single framework (10). The European Health Technology Assessment Regulation (Regulation (EU) 2021/2282; HTAR), now implemented and operational in the EU, also reflects this shift through its emphasis on ongoing evidence generation and reassessment, although the implications of this perspective shift for the assessment of digital health technologies and medical devices continue to require further methodological development. Early experiences of implementation for high-risk medical devices are already being reported (11).

An allied problem for HTA is how to facilitate early access pathways for DHTs addressing rare diseases, unmet needs, or health emergencies (12). In these situations, it may often be unrealistic or counterproductive to wait for high-quality comparative evidence before starting evaluation. A comprehensive framework detailing what type of evidence can be expected at each stage of device development would allow HTA bodies, payers, and health technology developers to make informed decisions based on the types of evidence that can realistically be expected at each stage in the life cycle.

## IDEAL as a conceptual foundation

The problem HTA faces is therefore how to transition from a single time-point evaluation to a more flexible approach that identifies and evaluates the evidence available at different stages of a technology’s life cycle. We propose a solution based on the IDEAL framework, an evidence-based methodology originally developed to improve the evaluation of surgical innovations (13). IDEAL is based on in-depth analysis of the life cycle of a procedure or device and identifies stages and specific questions that need to be answered at each stage. It outlines five key stages in the innovation trajectory: Idea, Development, Exploration, Assessment, and Long-term Study. For each stage, the evidence requirements are specified, and methodological guidance is offered, resulting in a structure that aligns evaluation with the maturity of the technology under consideration. Broadly, the Idea stage deals with preparation for and conduct of the first-in-human use of the intervention, the Development stage demonstrates that the device is stable in its performance, and the Exploration stage provides an estimate of effectiveness based on a large, heterogeneous, and representative population, and investigates issues that might bias a later RCT. Assessment represents the stage of comparative evaluation, which is the main focus of classical HTA, while long-term study equates roughly with post-market surveillance.

In the context of surgical procedures, IDEAL has helped to standardize reporting, improve transparency, and support proportionate evidence generation. Its principles have since been adapted for medical devices (IDEAL-D) (14), artificial intelligence-driven clinical systems (DECIDE-AI) (15), and surgical robots (16), demonstrating its versatility and relevance beyond the surgical domain. These adaptations demonstrate IDEAL’s utility in evaluating health technologies that require adaptation to the individual patient, operator skill, and iterative clinical development (17). The concept of life-cycle–based HTA frameworks has been proposed by several groups (2;5;12;18), which conceptualize HTA as a process spanning the full technology life cycle. LCBE builds on these foundations but extends life cycle thinking both to the earlier formative stages typical of digital health technologies and to surveillance mechanisms for DHTs that continue to modify themselves during ongoing clinical use, incorporating contextual modifiers, and risk stratification. While Kirwin et al. (2) provide the most developed existing parallel to this approach, their framework does not address the evaluation of stage-specific evidence gaps, an omission that risks overlooking the uncertainty and potential patient safety implications that accumulate when evidence requirements at earlier stages are not explicitly defined. The framework is consistent with broader European efforts to harmonize DHT assessment frameworks (19).

## Adapting IDEAL for digital health: the life-cycle–based evaluation model

Building on the IDEAL model, we are developing a Life-Cycle–Based Evaluation (LCBE) model for HTA evaluation tailored specifically for DHTs. This model ensures that evaluation is both continuous and proportionate. It recognizes that studies in early stages are necessarily formative or exploratory, whereas in later stages more rigorous, generalizable, and comparative evidence will be available. The earlier stages in the life cycle will inevitably lack randomized trials or cost–benefit analyses, while ongoing surveillance will be needed to evaluate continuous modifications of DHTs. By matching evaluative goals with developmental stages, LCBE allows rational evaluation of existing evidence, facilitates identification of evidence gaps, supports proportionate evidence generation and earlier decision making (such as that supported through managed access pathways such as German DiGA (20) or the French PECAN (21)), and facilitates reevaluation as technologies evolve (22). Detailed analysis of the limited information available is helpful for payers who must make risk-based judgments on how early clinical evaluation can be supported. The LCBE model for DHTs includes five life-cycle stages analogous to the IDEAL stages: preclinical, experimental/shadow mode, early clinical, comparative, and surveillance.

We have developed the LCBE framework through extensive consultations with stakeholder group experts and IDEAL methodologists to define the stages of the DHT life cycle, informed by systematic literature reviews and sampling of relevant databases. We have collaborated with partners within the Consortium who developed a validated taxonomy of DHTs according to their principal clinical purpose, as there are clear differences in the evaluation methods depending on purpose. Following harmonization with consortium partners, six Medical Purposes were defined: Prevention, Monitoring, Prognosis, Diagnosis, Treatment (with sub-options of Recommendation and Conduct), and Disability Alleviation. Expert consultations and reviews using an IDEAL framework approach to identifying essential stage-specific evaluations (13) led us to identify six important contextual variables that could influence the assessment needed for a DHT. These included: incorporated AI, autonomous function, and remote usage – for example, telemedicine, interoperability with other DHTs, and human factors issues (23). We developed recommendations for their incorporation into evaluation at each life-cycle stage. To align HTA with regulatory oversight, we stratified risk levels using the definitions of risk class in the European Medical Device Regulation.

**Figure 1.**
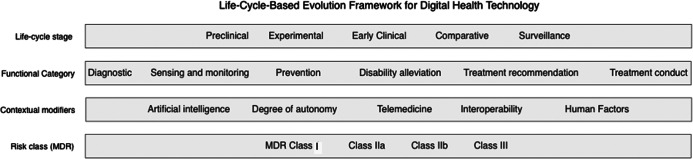
Life-cycle–based evaluation framework for digital health technologies. Figure 1. long description.

By combining life-cycle stage, technology function, risk, and contextual modifiers, the framework (see Figure 1) supports a wide range of stage-appropriate evaluation pathways. To demonstrate that this complexity can be managed in practice, we implemented the framework as a rule-based decision logic that generates tailored evaluation guidance from a small number of clearly defined technology characteristics. This operationalization serves to test the internal coherence and feasibility of the life-cycle approach, rather than as a prescriptive assessment tool.

## Delphi consensus process and stakeholder engagement

To test the coherence and validity of the life-cycle logic across stakeholder groups, we initiated a Delphi consensus process involving a diverse panel of experts from HTA, clinical practice, academia, industry, regulation, and patient advocacy. This exercise will ensure that the framework aligns with the requirements of real-world HTA and promotes “joined-up” activity by regulators, HTA, and payers through the development of a common language based on the life-cycle stages.

The Delphi process was completed in April 2026 and has informed further refinement of the LCBE framework; detailed results and methodology will be reported in subsequent publications. We thank the expert participants who contributed to the consensus process and whose input helped refine the framework. We recognize that HTA groups are already making efforts to adapt to the new challenges of DHTs and hope that the LCBE framework will be useful in unifying these.

## Conclusion

A key challenge for contemporary HTA is to ensure that real-world evidence is generated and analyzed in a way that is rigorous, transparent, and resilient to bias. The increasing complexity and dynamism of Digital Health Technologies require quite fundamental changes to the standard HTA model, as one-time, static evaluations fail to capture the evolving nature of these interventions and the conditions in which they operate. The LCBE model represents the first comprehensive attempt to provide a structured yet adaptable approach to evidence generation appropriate to the needs of DHTs across their entire life cycle, incorporating concerns commonly considered the separate domains of regulation and HTA, and unifying them in a common framework. We commend this approach because future frameworks for HTA will inevitably need to encompass a broader range of types of evidence and develop a staged approach to evaluation, thanks to evidence gaps in early stages and greatly increased plasticity and change throughout the life cycle. The LCBE framework will require further testing and iterative improvement, and we welcome expressions of interest from those interested in participating in this process.

Our goal is to contribute a flexible and comprehensive template for modern HTA, which will remain relevant in the face of continuous digital innovation, while maintaining the rigor necessary to ensure patient safety and system efficiency.

## Long descriptions

### Figure 1. Long description

A diagram titled ‘Life-Cycle-Based Evaluation Framework for Digital Health Technology’ comprising four labelled horizontal rows. Row 1, Life-cycle stage: Preclinical, Experimental, Early Clinical, Comparative, Surveillance. Row 2, Functional Category: Diagnostic, Sensing and monitoring, Prevention, Disability alleviation, Treatment recommendation, Treatment conduct. Row 3, Contextual modifiers: Artificial intelligence, Degree of autonomy, Telemedicine, Interoperability, Human Factors. Row 4, Risk class (MDR): MDR Class I, Class IIa, Class IIb, Class III.

Navigate back to Figure 1..

## References

[1] O’Rourke B, Oortwijn W, Schuller T, the International Joint Task Group. The new definition of health technology assessment: A milestone in international collaboration. Int J Technol Assess Health Care. 2020;36(3):187‐190. 10.1017/S0266462320000215. 32398176

[2] Kirwin E, Round J, Bond K, McCabe C. A conceptual framework for life-cycle health technology assessment. Value Health. 2022;25(7):1116‐1123. 10.1016/j.jval.2021.11.1373. 35779939

[3] Polisena J, Castaldo R, Ciani O, et al. Health technology assessment methods guidelines for medical devices: How can we address the gaps? The international federation of medical and biological engineering perspective. Int J Technol Assess Health Care. 2018;34:276‐289. 29909792 10.1017/S0266462318000314

[4] Ming J, He Y, Yang Y, et al. Health technology assessment of medical devices: Current landscape, challenges, and a way forward. Cost Eff Resour Alloc. 2022;20(1):54. 10.1186/s12962-022-00389-6. 36199144 PMC9533595

[5] Pichler FB, Boysen M, Mittmann N, et al. An operationalization framework for lifecycle health technology assessment: A health technology assessment international global policy forum task force report. Int J Technol Assess Health Care. 2024;40(1):e45. 10.1017/S0266462324000199. 38751240 PMC11569903

[6] NICE. NICE healthtech programme manual; 2025. p. 39. https://www.nice.org.uk/process/pmg48/resources/nice-healthtech-programme-manual-pdf-72286843070149

[7] Sahiner B, Chen W, Samala RK, Petrick N. Data drift in medical machine learning: Implications and potential remedies. Br J Radiol. 2023;96(1150):20220878. 10.1259/bjr.20220878. 36971405 PMC10546450

[8] Guo C, Ashrafian H, Ghafur S, et al. Challenges for the evaluation of digital health solutions—A call for innovative evidence generation approaches. npj Digit Med. 2020;3(1):110. 10.1038/s41746-020-00314-2. 32904379 PMC7453198

[9] Mathias R, McCulloch P, Chalkidou A, Gilbert S. How can regulation and reimbursement better accommodate flexible suites of digital health technologies? npj Digit Med. 2024;7(1):170. 10.1038/s41746-024-01156-y. 38956279 PMC11219713

[10] National Institute for Health and Care Excellence (NICE) and NHS England. AI and digital regulations service. 2025. Available from: https://www.digitalregulations.innovation.nhs.uk (accessed 24 Oct 2025).

[11] Alshaikh RA, Walsh KA, Spillane S, et al. The early experiences of health technology assessment bodies in the implementation of the European Union health technology assessment regulation for high-risk medical devices: A qualitative study. Value Health. 2025;29(6):1034‐1044. 41360323 10.1016/j.jval.2025.11.013

[12] Pichler FB, Boysen M, Mittmann N, et al. Lifecycle HTA: Promising applications and a framework for implementation. An HTAi global policy forum task force report. Int J Technol Assess Health Care. 2024;40(1):e50. 10.1017/S0266462324000187. 38727087 PMC11569898

[13] McCulloch P, Altman DG, Campbell WB, et al. No surgical innovation without evaluation: The IDEAL recommendations. Lancet. 2009;374(9695):1105‐1112. 10.1016/S0140-6736(09)61116-8. 19782876

[14] Sedrakyan A, Campbell B, Merino JG, et al. IDEAL-D: A rational framework for evaluating and regulating the use of medical devices. BMJ. 2016;353:i2372. https://pubmed.ncbi.nlm.nih.gov/27283585/27283585 10.1136/bmj.i2372

[15] Vasey B, Novak A, Ather S, Ibrahim M, McCulloch P. DECIDE-AI: A new reporting guideline and its relevance to artificial intelligence studies in radiology. Clin Radiol. 2023;78(2):130‐136. 10.1016/j.crad.2022.09.131. 36639172

[16] Marcus HJ, Ramirez PT, Khan DZ, et al. The IDEAL framework for surgical robotics: Development, comparative evaluation and long-term monitoring. Nat Med. 2024;30(1):61‐75. 10.1038/s41591-023-02732-7. 38242979 PMC13157720

[17] Alami H, Lehoux P, Auclair Y, et al. Artificial intelligence and health technology assessment: Anticipating a new level of complexity. J Med Internet Res. 2020;22(7):e17707. 10.2196/17707. 32406850 PMC7380986

[18] Thokala P, Srivastava T, Smith R, et al. Living health technology assessment: Issues, challenges and opportunities. PharmacoEconomics. 2023;41(3):227‐237. 10.1007/s40273-022-01229-4. 36652184 PMC9848020

[19] Tsiasiotis E, Mezei F, Di Bidino R, et al. Toward a harmonized health technology assessment framework for digital health technologies in Europe. Value Health. 2025;29(4):560‐566. 41443339 10.1016/j.jval.2025.12.004

[20] Sauermann S, Herzberg J, Burkert S, Habetha S. DiGA – A chance for the German healthcare system. Journal of European CME. 2022;11(1):2014047. 10.1080/21614083.2021.2014047. 34992948 PMC8725763

[21] Martin T, Hervias A, Armoiry X, Martelli N. Early access programs for medical devices in France: Overview of recent reforms and outcomes (2015–2022). Health Policy. 2024;148:105146. 10.1016/j.healthpol.2024.105146. 39154454

[22] Tarricone R, Ciani O, Torbica A, et al. Lifecycle evidence requirements for high-risk implantable medical devices: A European perspective. Expert Rev Med Devices. 2020;17(10):993‐1006. 10.1080/17434440.2020.1825074. 32975149

[23] Mathias R, Vasey B, Chalkidou A, et al. Safe AI-enabled digital health technologies need built-in open feedback. Nat Med. 2025;31(2):370‐375. 10.1038/s41591-024-03397-6. 39905271

